# Potential impact of 2018 Korean Society of Hypertension guidelines on Korean population: a population-based cohort study

**DOI:** 10.1186/s40885-020-0137-5

**Published:** 2020-02-01

**Authors:** Ju-Seung Kwun, Sun-Hwa Kim, Si-Hyuck Kang, Chang-Hwan Yoon, Hae-Young Lee, Kwang-Il Kim, Tae-Jin Youn, In-Ho Chae, Cheol-Ho Kim

**Affiliations:** 1 Cardiovascular Center, Department of Internal Medicine, Seoul National University Bundang Hospital, 82, Gumi-Ro 173 Beon-Gil, Bundang-Gu, Seongnam-Si, Gyeonggi-Do 13620 South Korea; 2 Department of Internal Medicine, Seoul National University, Seoul, South Korea; 3 Cardiovascular Center, Seoul National University Hospital, Seoul, South Korea

**Keywords:** Hypertension, Blood pressure, Korea National Health and nutrition examination survey (KNHANES), 2017 ACC/AHA guidelines, 2018 KSH guidelines

## Abstract

**Background:**

The Korean Society of Hypertension (KSH) revised the local guidelines for hypertension in 2018. The present study sought to evaluate the potential impact of the 2018 KSH guidelines on hypertension management status among the Korean population in terms of prevalence of hypertension, antihypertensive medical treatment recommendations, and control status in Korean adults.

**Methods:**

We used data from the Korea National Health and Nutrition Examination Survey to estimate the number and percentage of Korean adults who have hypertension according to blood pressure (BP) classification, are recommended to receive antihypertensive medical treatment, and are receiving medical treatment and have BP in the optimal range according to the new recommendations. Adults aged 30 years or older who participated in the survey between 2013 and 2015 were selected for this study.

**Results:**

The prevalence of hypertension was 30.5% among Korean adults aged 30 years or older. The percentage of subjects who are recommended to be treated with antihypertensive medications substantially increased from 32.5 to 37.8%, which translates to 1.6 million adults. Among the hypertensive patients who were receiving medical treatment, 38.6% were shown to have adequate BP levels as recommended by the 2018 KSH guidelines compared with 51.8% according to the previous 2013 guidelines.

**Conclusions:**

The present study reports the potential impact of the 2018 KSH guidelines on the prevalence of hypertension, antihypertensive medical treatment recommendations, and control status for Korean adults. The 2018 KSH guidelines recommend more intensive BP control compared with previous guidelines. This study suggests that there is large scope for improvement in hypertension management in the Korean population.

## Background

Elevated blood pressure (BP) is a leading global health risk factor [1, 2]. Elevated BP increases the risk of cardiovascular and renal events in a linear manner, and evidence indicates that the linear relationship extends down to 115/75 mmHg [3]. Recent studies have shown the benefits of intensive systolic BP control <130 mmHg in terms of adverse cardiovascular events and mortality [4].

The 2017 American College of Cardiology/American Heart Association (ACC/AHA) guidelines for hypertension lowered the hypertension criteria, BP threshold for medical treatment, and treatment goals [5]. A population-based study from the United States estimated that the prevalence of hypertension increased substantially, but the increase in the number of adults recommended to take antihypertensive medication was relatively small [6]. It was also shown that more intensive BP lowering would be required for many adults taking antihypertensive medication. A previous study showed that there should be a similar impact if the 2017 ACC/AHA guidelines were directly applied to the Korean population [7].

Recently, the Korean Society of Hypertension (KSH) updated the local guidelines for hypertension [8–10]. The criterion for hypertension was maintained as systolic BP ≥140 mmHg or diastolic BP ≥90 mmHg [8]. However, the new guidelines also support intensive BP control consistent with the 2017 ACC/AHA guidelines. Treatment threshold and BP target have been lowered for a variety of subgroups [9].

The present study was designed to estimate the potential impact of the 2018 guidelines for hypertension on the Korean population in terms of prevalence of hypertension, antihypertensive medical treatment recommendations, and control status in Korean adults. We aimed to report the number and percentage of Korean adults who have hypertension according to the BP classification, are recommended to receive antihypertensive medical treatment, and are receiving medical treatment and have BP in the optimal range according to the new recommendations.

## Methods

### Aim

The present study sought to evaluate the potential impact of the 2018 KSH guidelines on hypertension management status among the Korean population in terms of prevalence of hypertension, antihypertensive medical treatment recommendations, and control status for Korean adults.

### Data source and study population

Data from the Korea National Health and Nutrition Examination Survey (KNHANES) were used for the study purpose. KNHANES is a nationally representative surveillance system that assesses the health and nutritional status of Koreans [11]. The surveys are conducted by the Korea Centers for Disease Control and Prevention. Approximately 10,000 individuals are included each year, and data on socioeconomic status, health-related behaviors, quality of life, healthcare utilization, anthropometric measures, biochemical and clinical profiles for non-communicable diseases and dietary intakes with three component surveys are collected [11]. In the present study, adults aged 30 years or older were chosen among the participants in the KNHANES between 2013 and 2015.

### BP measurement

BP was measured by four nurses who underwent specialized training and whose performance was regularly monitored and certified. A mercury sphygmomanometer (Baumanometer; WA Baum Co., New York, NY, USA) was used for BP measurements. Study subjects were recommended to rest in a seated position for at least 5 min. BP was measured three times on the subjects’ right arms using an appropriately sized arm cuff. The average of the second and third measurements was used for data analysis.

### Definition

Table 1 summarizes the similarities and differences in BP classification between the 2018 and 2013 guidelines. The 2018 KSH guidelines recommended pharmacological treatment for BP ≥130/80 mmHg if a subject has three or more cardiovascular risk factors or subclinical organ damage. More strict BP control was recommended compared to the previous version for subjects with cardiovascular diseases or diabetes and adults aged more than 65 years. BP was classified as normal, elevated, and hypertension (grades 1 and 2) according to the 2018 KSH hypertension guidelines. Hypertension was defined as systolic BP ≥140 mmHg or diastolic BP ≥90 mmHg, and those already taking antihypertensive medications were also regarded as having hypertension. The number and percentage of adults requiring antihypertensive drug treatment were presented according to the guidelines. Control rate was defined as the proportion of adults for whom the recommended BP goals were achieved among those who were receiving hypertension treatment.

**Table 1:** Brief comparison of the 2018 Korean Hypertension guidelines and 2013 Korean Hypertension guidelines

	2018 Korean Hypertension guidelines	2013 Korean Hypertension guidelines
Definition of hypertension	SBP ≥140 mmHg or DBP ≥90 mmHg	SBP ≥140 mmHg or DBP ≥90 mmHg
Recommended pharmacologic treatment	- General population: SBP ≥140 mmHg or DBP ≥90 mmHg - DM, CKD, CVD: SBP ≥130 mmHg or DBP ≥80 mmHg - Presence ≥3 of CVD risk factors or subclinical organ damage: SBP ≥130 mmHg or DBP ≥80 mmHg	- General population: SBP ≥140 mmHg and DBP ≥90 mmHg - DM, CKD, CVD: SBP ≥130 mmHg or DBP ≥80 mmHg
Target BP goal	- General population: SBP < 140 mmHg and DBP < 90 mmHg - DM without CVD: SBP < 140 mmHg and DBP < 85 mmHg - DM with CVD: SBP < 130 mmHg and DBP < 80 mmHg - Stroke: SBP < 140 mmHg and DBP < 90 mmHg - Presence ≥3 risk factors: SBP < 130 mmHg and DBP < 80 mmHg - CVD: SBP < 130 mmHg and DBP < 80 mmHg - CKD without albuminuria: SBP < 140 mmHg and DBP < 90 mmHg - CKD with albuminuria: SBP < 130 mmHg and DBP < 80 mmHg - Elderly (≥65 years of age without DM or CKD): SBP < 140 mmHg and DBP < 90 mmHg	- General population: SBP < 140 mmHg and DBP < 90 mmHg - DM: SBP < 140 mmHg and DBP < 85 mmHg - Stroke: SBP < 140 mmHg and DBP < 90 mmHg - CAD: SBP < 140 mmHg and DBP < 90 mmHg - CKD without albuminuria: SBP < 140 mmHg and DBP < 90 mmHg - CKD with albuminuria: SBP < 130 mmHg and DBP < 80 mmHg - Elderly (≥65 years of age without DM or CKD): SBP < 150 mmHg and DBP < 90 mmHg

*SBP* systolic blood pressure, *DBP* diastolic blood pressure, *DM* diabetes mellitus; *CKD* chronic kidney disease, *CVD* cardiovascular disease, *CAD* coronary artery disease

### Statistical analysis

Data are presented as mean ± standard error or % (standard error). Weights based on the complex sampling design of KNHANES were used for all the statistical analyses to avoid biased estimates [11]. The variables representing strata, cluster, and weight were included in the raw data. The number and proportion of BP classification, subjects recommended for medical treatment, and those under control were estimated by weighted means. Statistical analyses were conducted using R programming version 3.2.4 (http://www.R-project.org; The R Foundation for Statistical Computing, Vienna, Austria).

## Results

### Prevalence of hypertension

**Fig. 1:**
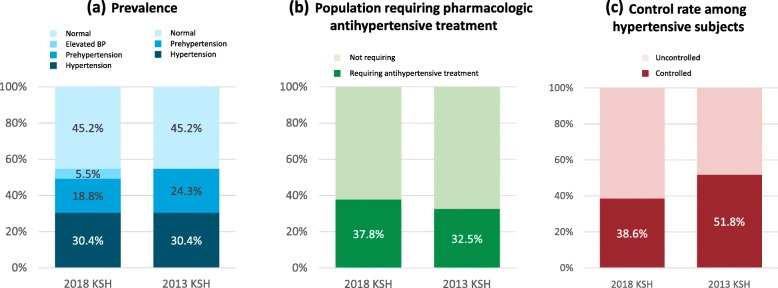
Comparison of 2018 and 2013 KSH guidelines. Prevalence of hypertension (**a**), proportion of adults recommended to receive pharmacological antihypertensive medication (**b**), and control rate below the target BP goal (**c**) among Korean adults according to the 2018 and 2013 KSH guidelines. BP = blood pressure, KSH=Korean Society of Hypertension.

A total of 9.1 million Korean adults (30.5%) were estimated to have hypertension (Fig. 1a). Approximately two-thirds were already receiving medical treatment, and the remaining one third were not. The prevalence of hypertension was higher among older adults, men and those living in rural areas (Additional file 1). Prehypertension accounted for 5.6 million adults (18.8%), whereas 1.7 million (5.5%) who had been regarded as having prehypertension according to the 2013 guidelines were classified as having elevated BP according to the new guideline.

The characteristics of the subjects in each BP category are described in Table 2. Compared to adults with hypertension, those with prehypertension were relatively younger and had lower prevalence of obesity and comorbidities. The estimated 10-year cardiovascular risk of normal BP groups was lower than hypertension groups. In contrast, adults with elevated BP were older than normal BP, grade 1 or grade 2 hypertension groups and their estimated cardiovascular risk was comparable to grade 1 hypertension.

**Table 2:** Subject characteristics according to blood pressure (BP) category and the use of antihypertensive medications

	Systolic/diastolic BP (mm Hg) categories with no antihypertensive medication					Taking antihypertensive medication
	Normal (< 120/80)	Elevated BP (120–129/< 80)	Prehypertension (130–139/80–89)	Grade 1 hypertension (140–159/90–99)	Grade 2 hypertension (≥160/100)	
Proportion (%)	45.2%	5.5%	18.8%	8.7%	2.1%	19.6%
Estimated number (million)	13.5	1.7	5.6	2.6	0.6	5.9
Age, years	45.9 ± 0.2	57.1 ± 0.6	49.0 ± 0.3	52.7 ± 0.8	51.4 ± 0.4	63.8 ± 0.3
Sex, %						
Men	38.7 ± 0.6	50.5 ± 2.1	61.0 ± 1.1	65.4 ± 1.6	64.6 ± 3.0	46.4 ± 1.0
Women	61.3 ± 0.6	49.5 ± 2.1	39.0 ± 1.1	34.6 ± 1.6	35.4 ± 3.0	53.6 ± 1.0
Anthropometric measurements						
Body mass index, kg/m^2^	23.0 ± 0.0	23.9 ± 0.1	24.5 ± 0.1	24.9 ± 0.1	26.0 ± 0.3	25.3 ± 0.1
Waist circumference, cm	78.8 ± 0.2	82.8 ± 0.4	83.6 ± 0.2	85.2 ± 0.3	87.1 ± 0.7	86.8 ± 0.2
Systolic BP, mmHg	105.5 ± 0.1	123.5 ± 0.1	123.1 ± 0.2	138.2 ± 0.4	158.7 ± 1.0	128.6 ± 0.4
Diastolic BP, mmHg	69.4 ± 0.1	73.1 ± 0.2	82.5 ± 0.1	88.6 ± 0.3	100.8 ± 0.8	76.3 ± 0.3
Heart rate, beat / min	56.9 ± 0.6	56.9 ± 1.6	57.3 ± 1.3	55.3 ± 0.5	71.5 ± 8.3	58.4 ± 0.8
Lifestyle behaviors						
Regular physical activity, %	33.5 ± 1.4	31.1 ± 3.5	35.2 ± 1.9	38.4 ± 3.0	42.4 ± 5.8	34.5 ± 1.6
Heavy alcohol drinking, %	11.4 ± 0.6	19.4 ± 2.2	20.9 ± 1.1	29.2 ± 1.9	37.0 ± 3.8	18.2 ± 1.2
Daily calorie intake, kcal	2045.0 ± 15.5	2046.0 ± 44.2	2197.7 ± 28.3	2159.6 ± 38.6	2317.3 ± 90.0	1822.3 ± 19.3
Daily sodium consumption, mg	4022.9 ± 46.8	4013.1 ± 139.6	4373.0 ± 109.5	4210.9 ± 103.9	4492.7 ± 215.2	3468.7 ± 58.6
Comorbidities, %						
Obesity	21.9 ± 0.7	34.1 ± 2.0	39.2 ± 1.2	47.0 ± 1.7	56.6 ± 3.5	50.7 ± 1.0
Diabetes mellitus	5.2 ± 0.4	15.0 ± 1.4	9.5 ± 0.7	10.8 ± 1.1	8.0 ± 2.0	27.6 ± 1.1
Dyslipidemia	62.7 ± 1.7	77.6 ± 2.4	75.1 ± 1.6	81.0 ± 1.8	75.6 ± 3.7	85.3 ± 1.1
Laboratory findings						
Total cholesterol, mg/dL	188.5 ± 0.5	193.7 ± 1.6	198.8 ± 0.9	201.4 ± 1.6	201.6 ± 2.4	185.3 ± 0.8
HDL cholesterol, mg/dL	52.1 ± 0.2	49.9 ± 0.5	49.8 ± 0.3	49.8 ± 0.4	48.8 ± 0.8	47.8 ± 0.3
LDL cholesterol, mg/dL	115.8 ± 0.7	117.1 ± 2.3	112.3 ± 1.2	119.7 ± 1.8	121.7 ± 3.1	107.8 ± 1.1
Triglyceride, mg/dL	121.9 ± 1.7	153.1 ± 5.7	161.6 ± 3.3	185.6 ± 5.8	196.8 ± 11.2	159.1 ± 2.8
Fasting glucose, mg/dL	95.9 ± 0.3	104.4 ± 1.0	102.1 ± 0.6	104.0 ± 0.9	104.9 ± 1.8	109.7 ± 0.6
Calculated GFR (mL/min/1.73m^2^)	95.8 ± 0.3	92.7 ± 0.8	93.5 ± 0.4	92.2 ± 0.6	92.2 ± 1.2	84.6 ± 0.4
10-year ASCVD risk	3.4 ± 0.1	10.7 ± 0.5	6.6 ± 0.2	10.7 ± 0.4	12.3 ± 0.8	19.6 ± 0.4
History of cardiovascular disease	1.9 ± 0.2	4.9 ± 0.8	2.3 ± 0.3	2.7 ± 0.5	1.1 ± 0.5	12.9 ± 0.7

Values were presented as mean ± SD or percent with numbers of subjects

*HDL* High density lipoprotein, *LDL* Low density lipoprotein, *GFR* Glomerular filtration rate, *ASCVD* Atherosclerotic cardiovascular disease

### Potential impact on pharmacological antihypertensive treatment

As shown in Table 1, the 2018 KSH guidelines expanded the indication for the pharmacologic treatment to prehypertensive subjects who are at high risk. The percentage of subjects who were recommended antihypertensive medical treatment increased from 32.5 to 37.8% (Fig. 1b), which is translated into 1.6 million adults (Table 3). The increase was numerically greater for men and middle-aged adults: three fourths of the increase were among men, and those between 40 and 69 years. Accordingly, the treatment rate among those indicated for medical treatment decreased from 60.4 to 52.0%.

**Table 3:** Percentage and number of Korean adults recommended antihypertensive medical treatment

	Percentage			Number		
	2018 KSH guidelines	2013 KSH guidelines	Difference	2018 KSH guidelines	2013 KSH guidelines	Difference
Total	37.8 ± 0.6	32.5 ± 0.6	5.3 ± 0.2	11.3	9.7	1.6
Sex						
Male	45.1 ± 0.8	36.6 ± 0.8	8.5 ± 0.5	6.4	5.2	1.2
Female	31.1 ± 0.7	28.7 ± 0.7	2.3 ± 0.2	4.8	4.5	0.4
Age (years)						
30–39	12.8 ± 0.8	9.5 ± 0.7	3.3 ± 0.4	0.9	0.7	0.2
40–49	27.2 ± 0.9	21.5 ± 0.9	5.7 ± 0.5	2.1	1.6	0.4
50–59	45.2 ± 1.1	36.4 ± 1.1	8.8 ± 0.6	3.3	2.6	0.6
60–69	57.9 ± 1.2	52.9 ± 1.2	5.1 ± 0.5	2.5	2.3	0.2
70+	68.2 ± 1.2	66.3 ± 1.2	1.8 ± 0.3	2.6	2.5	0.1
Area of residence						
Urban area	36.7 ± 0.7	31.6 ± 0.6	5.1 ± 0.3	8.9	7.7	1.2
Rural area	42.7 ± 1.4	36.5 ± 1.4	6.2 ± 0.5	2.4	2.0	0.3
Income quartiles						
Highest	35.7 ± 1.1	30.5 ± 1.0	5.3 ± 0.5	2.7	2.3	0.4
Upper middle	36.3 ± 1.0	31.3 ± 1.0	5.0 ± 0.4	2.7	2.3	0.4
Lower middle	39.5 ± 1.1	33.4 ± 1.0	6.2 ± 0.5	3.0	2.5	0.5
Lowest	39.6 ± 1.0	35.0 ± 1.0	4.6 ± 0.5	2.9	2.5	0.3
Education levels						
Primary	62.1 ± 1.0	57.3 ± 1.1	4.8 ± 0.5	3.8	3.5	0.3
Middle	47.8 ± 1.5	41.7 ± 1.4	6.2 ± 0.7	1.5	1.3	0.2
High	32.7 ± 0.9	27.3 ± 0.8	5.4 ± 0.4	3.2	2.7	0.5
University/college	24.5 ± 0.8	19.2 ± 0.8	5.3 ± 0.4	2.5	2.0	0.5

Data are presented as mean ± SE

*KSH* Korean Society of Hypertension

### Potential impact on control rate

In the 2018 KSH guidelines, a strict BP target is recommended for several clinical situations such as the presence of cardiovascular disease and multiple risk factors. Among the adults who were receiving medical treatment, 38.6% were shown to have adequate BP levels recommended by the 2018 KSH guidelines compared with 51.8% according to the 2013 guidelines (Fig. 1c) (Table 4). The control rate was numerically low among men and younger people. Additional file 2 presents the difference in control rate according to specific conditions, which shows that tighter BP control is required especially among those with cardiovascular disease, stroke, and advanced age.

**Table 4:** Proportion and number of subjects with hypertension whose blood pressure is controlled under recommended levels

	Proportion			Number		
	2018 KSH guidelines	2013 KSH guidelines	Difference	2018 KSH guidelines	2013 KSH guidelines	Difference
Total	38.6 ± 1.0	51.8 ± 1.1	13.2 ± 0.6	2.8	3.7	1.0
Sex						
Male	32.1 ± 1.3	45.1 ± 1.5	13.0 ± 0.9	1.3	1.8	0.5
Female	46.7 ± 1.4	60.2 ± 1.3	13.5 ± 0.9	1.5	1.9	0.4
Age (years)						
30–39	8.3 ± 2.3	11.6 ± 2.7	3.3 ± 1.6	0.0	0.1	0.0
40–49	20.2 ± 2.2	30.9 ± 2.8	10.7 ± 1.8	0.2	0.3	0.1
50–59	29.1 ± 2.0	41.2 ± 2.1	12.2 ± 1.3	0.6	0.8	0.2
60–69	52.4 ± 1.7	66.6 ± 1.6	14.2 ± 1.2	1.0	1.2	0.3
70+	53.6 ± 1.7	70.9 ± 1.5	17.3 ± 1.2	1.0	1.3	0.3
Area of residence						
Urban area	38.2 ± 1.2	51.2 ± 1.2	13.0 ± 0.7	2.2	2.9	0.7
Rural area	39.9 ± 2.3	54.0 ± 2.5	14.1 ± 1.4	0.6	0.8	0.2
Income quartiles						
Highest	40.5 ± 2.0	54.8 ± 2.1	14.3 ± 1.4	0.7	1.0	0.3
Upper middle	37.9 ± 1.9	50.0 ± 2.0	12.1 ± 1.2	0.7	0.9	0.2
Lower middle	36.4 ± 1.9	48.9 ± 2.1	12.5 ± 1.2	0.7	0.9	0.2
Lowest	39.2 ± 1.8	53.3 ± 1.9	14.1 ± 1.3	0.7	1.0	0.3
Education levels						
Primary	46.7 ± 1.4	62.4 ± 1.4	15.8 ± 1.0	1.3	1.7	0.4
Middle	41.1 ± 2.4	54.2 ± 2.6	13.0 ± 1.6	0.4	0.5	0.1
High	36.5 ± 1.9	48.4 ± 2.0	12.0 ± 1.2	0.7	1.0	0.2
University/college	24.7 ± 2.0	35.0 ± 2.3	10.3 ± 1.4	0.4	0.5	0.2

Data are presented as mean ± SE

*KSH* Korean Society of Hypertension

## Discussion

The present study estimated the potential impact of the 2018 KSH guidelines on the prevalence of hypertension, antihypertensive medical treatment recommendation, and control status in Korean adults. The guidelines maintained the definition criteria for hypertension, and thus, the prevalence of hypertension in Korea remained at 30.5%. However, as more intensive BP control is recommended in the new guideline, there was a significant increase in the number of adults requiring antihypertensive medication. The proportion increased from 32.5 to 37.8%, which was responsible for 1.6 million Koreans. The rate of hypertensive patients whose BP was below recommended levels also decreased.

The 2018 KSH guidelines maintained its BP criteria for hypertension (systolic BP ≥140 mmHg or diastolic BP ≥90 mmHg) [8]. There were only small changes in the ranges of elevated BP and prehypertension [12]. The 2017 ACC/AHA hypertension guidelines lowered the threshold for hypertension from 140/90 mmHg to 130/80 mmHg [13]. A study by Muntner et al. estimated the potential impact of the guidelines changes in U.S. adults, and expected a substantial increase in the prevalence of hypertension (from 31.9 to 45.6%) [6].

However, the two guidelines are in a line that they both recommend intensive BP control [9, 10, 13]. The study by Muntner et al. estimated that the proportion of U.S. adults recommended to receive medical treatment would increase by 1.9% (from 34.3 to 36.2%) [6]. As shown in this study, the number of Korean adults who meet the criteria for medical treatment increased remarkably (from 32.5 to 37.8%). In a previous study, we showed that 35.3% of Korean adults would be recommended to receive antihypertensive medication when the 2017 ACC/AHA guidelines were directly applied the Korean population. Although the 2018 KSH guidelines maintained the criteria for hypertension the adoption of more intensive BP control was consistent with the 2017 ACC/AHA guidelines. As a result, antihypertensive medical treatment is recommended for a significant proportion of Korean adults who are classified as having prehypertension.

The changes are also reflected in the decrease in the control rate. The target BP for uncomplicated general hypertensive adults was maintained at <140/90 mmHg. However, as shown in Table 1, the target goal had been lowered for various subgroups compared with the 2013 version. For example, the 2013 KSH recommended target systolic BP to be approximately 140 to 150 mmHg. In contrast, the 2018 KSH guidelines stated that the effect of drug therapy against hypertension is clear irrespective of age, and recommended a target BP <140/90 mmHg in elderly hypertensive patients. In addition, the BP target goal for patients with complicated diabetes, high-risk profile, and cardiovascular disease had been lowered to <130/80 mmHg. More intensive BP control would be necessary for those individuals to achieve the new BP goals.

The transitions are based on recent trial results. The SPRINT trial enrolled patients with systolic BP ≥130 mmHg and a history of coronary artery disease, peripheral vascular disease, aortic disease, heart failure or left ventricular hypertrophy. The intensive strategy of lowering systolic BP < 120 mmHg resulted in significantly lower rates of fatal and non-fatal major cardiovascular events compared to the conventional strategy of systolic BP < 140 mmHg [4]. Meta-analyses also indicated that systolic BP < 130 mmHg reduces major cardiovascular outcomes including stroke, coronary events, heart failure, and cardiovascular mortality compared with >130 mmHg [14–16]. The move toward intensive BP control is also similar in the U.S. and in Europe [5, 17]. Previous studies have shown hypertension awareness, treatment, and control rates increased remarkably until 2007, but showed a plateau thereafter [12, 18]. The present study also suggests that there is room for improvement in BP control among Korean adults with hypertension.

One interesting finding in the present study is the relatively high-risk profile of the “elevated BP” group (systolic BP of 120–129 mmHg and diastolic BP <80 mmHg). In the study by Muntner et al., which analyzed the U.S. population, the level of risk in this group was between those of the “normal” (<120/80 mmHg) and “stage 1 hypertension” (130–139/80–89 mmHg) groups [6]. In the present study, their expected 10-year risk was higher than that of prehypertension (130–139/80–89 mmHg), and comparable to that of grade 1 hypertension (140–159/90–99 mmHg). The key factor was age. The mean age of this group was even older than the age of those with grade 1 or 2 hypertension. This is consistent with the observations that pulse pressure (systolic BP minus diastolic BP) increases with age, and that increased pulse pressure is associated with higher risk of future cardiovascular events [19, 20]. Subjects in this group are currently not candidates for antihypertensive treatment. The possibility of masked hypertension needs to be ruled out [21]. In addition, primary prevention such as lifestyle modification and cholesterol lowering should be considered for this group.

The major limitation of the present study originates from the data source. KNHANES is a nationally representative database that enrolls approximately 10,000 subjects living in Korea who were chosen by a complex stratified survey method. The use of KNHANES is the best way to estimate the impact in terms of the general Korean population. However, BP was measured during a single visit and a mercury sphygmomanometer was used by trained nurses. Recent evidence suggests unattended office BP measurement, ambulatory BP monitoring, and home BP monitoring may provide better estimates compared with conventional BP measurements [22–24]. In addition, while the presence of subclinical organ damages is used as one of the criteria for antihypertensive medical treatment in the 2018 KSH guideline, they were not available from the database.

## Conclusions

The present study revealed that the 2018 KSH guidelines for hypertension compared to the 2013 version, recommend more intensive BP control in the Korean population. We found that would be a remarkable increase in the number of adults who are recommended to receive medical treatment, and a decline in the hypertension control rate. This study suggests that there is a large scope for improvement in BP control in Korean adults.

## Supplementary information

**Additional file 1: Table S1.** Prevalence of hypertension in Korea (2013–2015).

**Additional file 2: Table S2.** Hypertension control rate according to specific clinical conditions.

## Data Availability

The data that support the findings of this study are available from the website (http://knhanes.cdc.go.kr). But restrictions apply to the availability of these data, which were used under license for the current study, and so are not publicly available.

## Abbreviations

ACC/AHA: American College of Cardiology/American Heart Association
BP: Blood pressure
KNHANES: Korea National Health and Nutrition Examination Survey
KSH: Korean Society of Hypertension
