# Supplementary material for: Potential impact of 2018 Korean Society of Hypertension guidelines on Korean population: a population-based cohort study
Source: Clin Hypertens. 2020 Feb 1;26:3. doi: 10.1186/s40885-020-0137-5 (PMC6995175; doi:10.1186/s40885-020-0137-5)
Supplement: Supplementary file 1 — Additional file 1: Table S1. Prevalence of hypertension in Korea (2013–2015). [file 40885_2020_137_MOESM1_ESM.docx]

**Supplementary Table 1.** Prevalence of hypertension in Korea (2013‒2015)

|  | Prevalence | |
| --- | --- | --- |
|  | Percentage (%) | Number (million) |
| Total | 30.5 ± 0.6 | 9.1 |
| Sex |  |  |
| Male | 33.7 ± 0.8 | 4.8 |
| Female | 27.4 ± 0.6 | 4.3 |
| Age (years) |  |  |
| 30-39 | 8.6 ± 0.6 | 0.6 |
| 40-49 | 19.4 ± 0.9 | 1.5 |
| 50-59 | 34.0 ± 1.0 | 2.4 |
| 60-69 | 49.7 ± 1.2 | 2.1 |
| 70+ | 64.5 ± 1.2 | 2.4 |
| Area of residence |  |  |
| Urban area | 29.5 ± 0.6 | 7.2 |
| Rural area | 34.5 ± 1.4 | 1.9 |
| Income quartiles |  |  |
| Highest | 28.7 ± 1.0 | 2.1 |
| Upper middle | 29.5 ± 0.9 | 2.2 |
| Lower middle | 31.3 ± 1.0 | 2.3 |
| Lowest | 32.4 ± 1.0 | 2.4 |
| Education levels |  |  |
| Primary | 80.2 ± 1.0 | 3.4 |
| Middle | 69.2 ± 2.1 | 1.2 |
| High | 62.0 ± 1.8 | 2.5 |
| University/college | 48.5 ± 2.3 | 1.8 |

Data are presented as mean ± SE.
