# Supplementary material for: Potential impact of 2018 Korean Society of Hypertension guidelines on Korean population: a population-based cohort study
Source: Clin Hypertens. 2020 Feb 1;26:3. doi: 10.1186/s40885-020-0137-5 (PMC6995175; doi:10.1186/s40885-020-0137-5)
Supplement: Supplementary file 2 — Additional file 2: Table S2. Hypertension control rate according to specific clinical conditions. [file 40885_2020_137_MOESM2_ESM.docx]

**Supplementary Table 2.** Hypertension control rate according to specific clinical conditions

|  | 2018 KSH guideline (%) | 2013 KSH guideline (%) | Difference (%) |
| --- | --- | --- | --- |
| Total | 38.6 ± 1.0 | 51.8 ± 1.1 | 13.2 ± 0.6 |
| Cardiovascular disease | 41.5 ± 2.8 | 63.2 ± 2.7 | 21.7 ± 2.2 |
| Diabetes |  |  |  |
| Diabetes without cardiovascular disease | 51.3 ± 2.1 | 56.7 ± 2.1 | 5.4 ± 1.0 |
| Diabetes with cardiovascular disease | 39.2 ± 4.6 | 60.6 ± 4.9 | 21.3 ± 3.7 |
| Age ≥65 years | 55.4 ± 1.3 | 71.6 ± 1.2 | 16.2 ± 1.0 |
| Risk factors ≥3 | 37.0 ± 1.2 | 52.7 ± 1.3 | 15.7 ± 1.0 |
| Stroke | 41.9 ± 4.1 | 60.4 ± 4.3 | 18.5 ± 3.1 |
| Chronic kidney disease |  |  |  |
| Albumin-creatinine ratio <30 | 60.3 ± 4.3 | 75.5 ± 3.9 | 15.2 ± 3.0 |
| Albumin-creatinine ratio ≥30 | 23.3 ± 6.4 | 27.6 ± 6.8 | 4.3 ± 2.6 |

Data are presented as mean ± SE.
